# Transdisciplinary team science: a field study in understanding and improving complex scientific problem solving

**DOI:** 10.3389/fpsyg.2026.1804489

**Published:** 2026-10-26

**Authors:** Stephen M. Fiore, Deborah DiazGranados, Troy Hartley

**Affiliations:** 1 University of Central Florida, Orlando, FL, United States; 2 Virginia Commonwealth University, Richmond, VA, United States; 3 Renewable Energy Wildlife Institute, Washington, DC, United States

**Keywords:** conceptual framework, science of team science, team reflexivity, team science, team training, transdisciplinarity

## Abstract

**Introduction:**

Transdisciplinary team science requires foundational teamwork competencies that support complex collaborative problem solving. This study examined the development of these competencies among graduate students from different disciplines.

**Methods:**

We conducted a field study of a training program grounded in foundational competencies for scientific teamwork. We examined participants’ self-reported collaboration competencies and the relationship between their teamwork self-ratings and independent expert evaluations of their scientific output.

**Results:**

Participants reported improved collaboration competencies following the training program. Their teamwork self-ratings also correlated with independent expert evaluations of their scientific output.

**Discussion:**

These findings suggest the value of competency-based training for transdisciplinary scientific teams and connect participants’ perceptions of teamwork with independently evaluated scientific output. They inform the development and testing of team science principles and approaches to studying complex collaborative problem solving.

## 1 Introduction

Addressing modern scientific and societal problems calls for research collaborations that extend across disciplinary, institutional, and geographic boundaries. Ongoing advances in technology, increasing specialization, and the rise of international initiatives have created new possibilities for innovation while also complicating coordination among scientists. Team science has emerged as an essential framework for supporting more complex research initiatives (Fiore, 2008; Hall et al., 2008; Stokols et al., 2008; Wuchty et al., 2007). This enhances how research operates by working across social, organizational, and technological systems (e.g., Börner et al., 2010).

The challenge is that realizing the full potential of team science requires intentional, evidence-based practices to enhance team processes, as well as institutional incentives and federal policy that provide a supportive context. The Science of Team Science (SciTS) field emerged to meet this need by systematically investigating the factors that promote successful scientific collaboration (Hall et al., 2008; Stokols et al., 2008). SciTS is a cross-disciplinary field created to improve the understanding and effectiveness of scientific collaboration. It focuses on developing an evidence base for enhancing team science effectiveness (Hall et al., 2018).

At its core, SciTS seeks to generate empirical understanding of the multiple, interacting influences on team science. This can be in broader areas like science policy (Falk-Krzesinski et al., 2011), attend to more contextual factors like organizational dynamics (e.g., Cummings and Kiesler, 2014), or consider the psychological dimensions of scientific collaboration (e.g., Fiore, 2008). The field draws on and extends theories, frameworks, and methods from a range of disciplines that study scientific practice and collaboration. From this, SciTS scholars have identified research examining the diverse factors that shape how science teams are formed and function as well as how various scientific products are related to different processes (National Academies of Sciences Committee on the Science of Team Science, 2015).

Despite progress in greater understanding of the challenges associated with scientific collaboration, there is still surprisingly little research on the science teams themselves and how they collaborate and how to help them become better scientific problem solvers (National Academies of Sciences Committee on the Science of Team Science, 2025). Effective scientific teamwork is challenging, and often not part of educational or professional training. Although many undergraduate and graduate programs include group projects as part of the coursework, few, if any, actually provide students any instruction on how to work effectively as a team. As such, this can mislead students into believing they are good at collaboration when they have only been evaluated on their group project outcome (Fiore et al., 2018). Learning how to collaborate requires deliberate interventions based on sound theory. Further, it requires opportunities for practice and targeted feedback. From this, learners can build and reflect on foundational competencies necessary for functioning within teams made up of members from varied disciplines.

We take this as our starting point and contribute to the growing body of literature on the SciTS. We describe a field study on transdisciplinary training for graduate students interested in improving their competencies in collaborative science in the marine sciences. Such research is crucial when considering grand societal challenges in complex natural and societal problems. In the marine context, transdisciplinary approaches are needed to address complex, coupled natural-human or socio-ecological systems, such as coastal oceans and estuaries. Building coastal resilience to climate change impacts is a particularly challenging problem that crosses natural and social sciences, engineering, design, public health, and other disciplinary boundaries, and demands highly integrated scientific analysis and solutions (Moser et al., 2012; Harris et al., 2010; Incropera, 2015). Similar to grand societal challenges, these natural problems are complex, with multiple feedback loops and they have high uncertainty along with ambiguous solutions (Rittel and Webber, 1973). Understanding and solving coastal resilience problems will push the capacity of coastal ocean and estuarine STEM fields and challenge the next generation of marine researchers to engage in all levels of cross-disciplinary research, and particularly transdisciplinary team science.

What makes such research difficult is that advanced cross-disciplinary solutions are difficult to achieve (Gray, 2008; Rhoten, 2003; Roy et al., 2013). In the area of solving natural scientific problems, researchers have identified several barriers (Palmer et al., 2016). Among the challenges were limitations in the key collaborative competencies needed to advance interdisciplinary teams (e.g., team management and relationship competencies). Similar research finds a lack of understanding or respect among different disciplines, power dynamics, and other problematic relations within teams, along with professional language differences interfering with development of shared understanding (e.g., Bammer et al., 2020; Pohl et al., 2021). Teams lacked cohesion among unfamiliar participants, lacked guidance or leadership, and exhibited uncertainty over goals and roles (Palmer et al., 2016).

What makes this form of scientific problem solving additionally complicated is human dependence on nature for their well-being (Potschin-Young et al., 2018). This requires managing ecosystems, which, itself, draws from governance systems supportive of people and the environment (e.g., Primmer et al., 2015). Because this requires balancing values about economic well-being with environmental and human health, conflict among stakeholders can sometimes occur (e.g., marine biologists worried about ocean preservation and businesses concerned about economic growth). This illustrates how social science disciplines are inextricably linked to sustainability research involving biodiversity and ecosystems (e.g., Spangenberg et al., 2014). Overall, this involves integration of knowledge about human and natural systems and governance of these systems supportive of sustainable outcomes (Chapman and Maher, 2014; Cordier et al., 2014).

In sum, challenges in marine science align with challenges for effective scientific collaboration among multiple disciplines tackling complex societal problems (e.g., Hoffmann et al., 2019; Zhang et al., 2025). These demand teamwork competencies helping scientists produce innovative breakthroughs that transcend individual disciplines, reaching a deeper level of knowledge integration (Fiore, 2008; Hall et al., 2008; NAS, 2015; Salazar et al., 2012; Stokols et al., 2008). Our field study set out to develop teamwork competencies for transdisciplinary science drawing from diverse disciplines related to marine science. In the next section we summarize the core concepts studied in our training and their origins in the organizational and cognitive sciences. Following this, we discuss how these are operationalized, connecting them to scientific teamwork at the interface of natural and social systems.

## 2 Foundational concepts for transdisciplinary science

In this section we first address the complementarity of teamwork and taskwork as competencies need for effective teams. We then discuss knowledge integration as a critical form of taskwork necessary for transdisciplinary science. And we then describe how reflexivity can be adapted as a way to improve teamwork competencies in science teams. Although these concepts have been independently studied in different settings, our field study was designed to examine how their combination can influence outcomes in real world scientific problem solving.

### 2.1 Distinguishing taskwork and teamwork

Central to team science is the distinction between taskwork and teamwork (Cannon-Bowers et al., 1995), originally developed to differentiate workplace training competencies (e.g., Rapp and Mathieu, 2007). Taskwork encompasses the domain-specific activities required to achieve project goals. In science, these include understanding theory, designing studies, applying methods, analyzing data, and disseminating findings. Teamwork encompasses the competencies required to coordinate interdependent collaborators. These include attitudinal competencies (e.g., trust), behavioral competencies (e.g., communication), and cognitive competencies (e.g., mental models of team roles and responsibilities).

Organizational research demonstrates that taskwork and teamwork provide distinct pathways for team development. Mathieu et al. (2000) found that taskwork and teamwork training promoted convergence in shared mental models of team and task roles and responsibilities. Fisher (2014) found that taskwork planning predicted coordination beyond teamwork planning, whereas teamwork planning predicted interpersonal processes beyond taskwork planning. More recently, Bendell et al. (2025) showed that generic teamwork aptitudes predicted overall team performance outcomes.

Fiore (2008) extended this distinction to cross-disciplinary scientific collaboration. Taskwork draws on disciplinary expertise, whereas teamwork enables collaborators to integrate knowledge across disciplines. Yet scientists are often underprepared for these collaborative demands because their education and professional development emphasize taskwork. This limited attention to teamwork processes creates a critical barrier to scientific success. Advancing team-based science therefore requires developing both taskwork and teamwork competencies (see also Fiore et al., 2015, 2019).

### 2.2 Knowledge integration in transdisciplinary teams

Knowledge integration is a foundational task-based competency in transdisciplinary research (e.g., Laursen, 2024; Pohl and Hadorn, 2008). It involves synthesizing theories, concepts, and methods from diverse fields to generate new insights (Pohl and Hadorn, 2008). At the team level, integration occurs when members combine distinct expertise into a new whole through social and cognitive processes that support communication, shared problem conceptualization, and recognition of how knowledge can be combined (Balakrishnan et al., 2011; Salazar et al., 2012).

More broadly, knowledge integration is viewed as a part of team learning; that is, as an open-ended, collaborative learning process that connects previously unrelated scientific and societal knowledge, ideas, and practices (Pohl et al., 2021). Laursen (2024) theorized different forms of integrative topologies (e.g., weaves, zippers, chains), depicting how varied types of integrative inputs, processes, and outputs can flexibly support collaboration across contexts. We adopt a transdisciplinary perspective that integrates diverse forms of expertise, including those of societal partners, to address complex real-world problems (Zhang et al., 2025).

Our study examines integration through conceptual frameworks developed in transdisciplinary research. We view these frameworks as cognitive artifacts, that is, physical or digital objects created to support cognitive and collaborative processes (Fiore and Wiltshire, 2016; Hollan et al., 2000; Hutchins, 1995; Norman, 1991). Such artifacts can be integral to teamwork in general (Cole and Engeström, 1993; Zhang and Patel, 2006) but are particularly useful for transdisciplinary teams because they help them make knowledge explicit, allowing discussion and debate across disciplinary boundaries (cf. Fiore et al., 2010; Zhang et al., 2025).

Conceptual frameworks organize key elements, relationships, and processes across disciplinary perspectives, providing a more comprehensive understanding of complex scientific problems (Bammer et al., 2020; Pohl et al., 2021). Prior models have connected contextual, technological, and organizational factors affecting how science teams study problems (Ghamgosar et al., 2023). Frameworks can also specify relationships among variables, identify potential interventions and measures (Rolland et al., 2021), and provide criteria for evaluating team-science initiatives (Knickel et al., 2019; Platt et al., 2024).

Framework development also serves an important collaborative function. By making diverse perspectives and assumptions visible, it can promote inclusiveness, transparency, and trust among team members from different backgrounds and positions within power hierarchies (cf. Scholz and Steiner, 2015; Zhang et al., 2025).

### 2.3 Teamwork reflection during team science

Team reflexivity is an important competency for team success. It involves members critically examining their collaborative experiences, evaluating performance, identifying improvements, and adapting future strategies. In scientific teams, reflexivity supports both learning how to improve teamwork while also improving team effectiveness.

Joint learning and reflection occur when team members draw on one another’s diverse backgrounds and experiences (Pennington et al., 2021). Participatory and co-creation methods extend this process by involving academic and community stakeholders in knowledge production (Tebes and Thai, 2018). For example, community members may participate in research design, data collection, and dissemination, enabling teams to consider the relevance and legitimacy of different perspectives (Bammer, 2019; Grigorovich et al., 2019). Scientists may also examine how disciplinary training shapes their assumptions and perceptions of other fields’ contributions (O’Rourke and Robinson, 2020).

Drawing on this research, our study uses a team reflexivity intervention focused on specific episodes of scientific problem solving. Effective team members recognize when an approach is not working and adjust their behavior accordingly (e.g., Gabelica et al., 2012; Lattuca, 2001; Lattuca et al., 2013, 2017; Stokols, 2014). Our intervention also incorporates collaborative reflection on feedback about team processes (O’Neill et al., 2018). Teams are more effective when they collectively examine their work and collaboration, then plan and implement changes (West, 2002; Schippers et al., 2003, 2014). Team-level performance feedback helps members connect their perceptions to collective performance and identify areas for improvement (e.g., Gabelica et al., 2012). Combining such feedback with reflection can strengthen team processes (Donia et al., 2018; Gabelica et al., 2014; Prins et al., 2006). This capacity is especially valuable in transdisciplinary research, where problem understanding evolves as teams learn more, and as goals develop over time based upon improved awareness of the problem. Our study uses reflection on a team’s prior collaboration to focus on facets on team processes. By providing teams with self-assessment feedback, they gain shared awareness about problematic processes and can target methods for improvement.

### 2.4 Summary: task integration and team reflection for scientific teamwork

The preceding sections distinguished teamwork from taskwork to clarify what scientists working across disciplines must learn. We identified knowledge integration as a central taskwork competency and conceptual framework development as one way transdisciplinary teams represent that integration. We also presented team reflexivity as a means of developing teamwork competence.

Although reflexivity has long been recognized as important (Guzzo, 1986; Hackman, 2002), teams do not naturally engage in regular, deliberate planning (Hackman and Morris, 1976; Weingart, 1992), and reflexivity interventions have not been systematically tested in science teams. In transdisciplinary research, reflection may strengthen teamwork while helping members develop a shared understanding of the problem and identify more integrative solutions (e.g., Gabelica et al., 2016). By improving communication about differing perspectives, reflection can help teams reconceptualize a domain or problem (Keestra, 2017).

Building on these concepts, we conducted a field study of transdisciplinary collaborative problem solving. Research proposals and conceptual frameworks served as indicators of taskwork and knowledge integration, while team-process measures assessed teamwork. Team reflection was the primary intervention for developing teamwork competence. It prompted members to examine their collaborative processes (e.g., information sharing), and one another’s theories, concepts, and methods. Together, these activities were intended to help teams integrate diverse disciplinary perspectives and develop a richer understanding of complex scientific problems while improving their teamwork competence.

### 2.5 Current study

We developed a workshop-based program combining synchronous collocated teamwork along with virtual synchronous and asynchronous collaboration. Workshops are often used as a form of professional development in science because they provide a focused form of training. These have been used to improve idea development for early career scientists (Gosselin et al., 2020) to improve engagement with alternative ways of thinking, to help improve transdisciplinary thinking for interdisciplinary scientists (e.g., Morgan et al., 2021; Pennington, 2016; Pennington et al., 2016, 2021), as well as to support ideation with scientists working to integrate knowledge in service of innovation (Hawk et al., 2024).

## 3 Methods

### 3.1 Participants and selection procedure

Participants were recruited as part of a graduate training professional development program funded by National Science Foundation’s Innovation in Graduate Education program. Participants were sought from across the United States to participate in a professional development opportunity. They were told that they would learn how to effectively work on teams of scientist from multiple disciplines and how to integrate diverse disciplinary perspectives to address the real-world challenge of enhancing resilience to climate change. Applicants had to be enrolled in a full-time graduate or professional degree program during their participation. The workshop cohort consisted of 36 graduate-level participants recruited from diverse institutions across the United States. To ensure a transdisciplinary approach, participants were selected from a wide range of academic backgrounds. The cohort was predominantly composed of doctoral candidates (61%), followed by master’s students (*n* = 33%) and law students (J.D. candidates, 6%). Last, 64% of participants were female and 36% were male.

The disciplinary composition was intentionally broad to facilitate cross-sector collaboration. The largest group, consisting of 50% of participants, represented the natural and marine sciences, (e.g., oceanography, marine ecology, fisheries, geology, and climatology). This was complemented with 28% coming from engineering and the built environment studies (landscape architecture, civil engineering, urban planning, and architecture). Finally, about 22% were participants from social sciences, law, and policy (public administration, environmental economics, and law), ensuring that discussions incorporated legal and regulatory frameworks alongside technical solutions.

### 3.2 Procedure

#### 3.2.1 Workshop preparation

Prior to the initial workshop, participants were emailed background information about an environmental resilience case developed in collaboration with Virginia Institute of Marine Sciences and local government officials. Participants were instructed to review the case material and develop a conceptual framework from their own disciplinary perspective. Instructions were provided on the meaning of a conceptual framework and that they were to conceive of how they would conduct research on their case and represent as a framework. Participants were instructed to complete these prior to the first workshop and bring with them to share with team members. Figure 1 provides an overview of the study.

**Figure 1:**
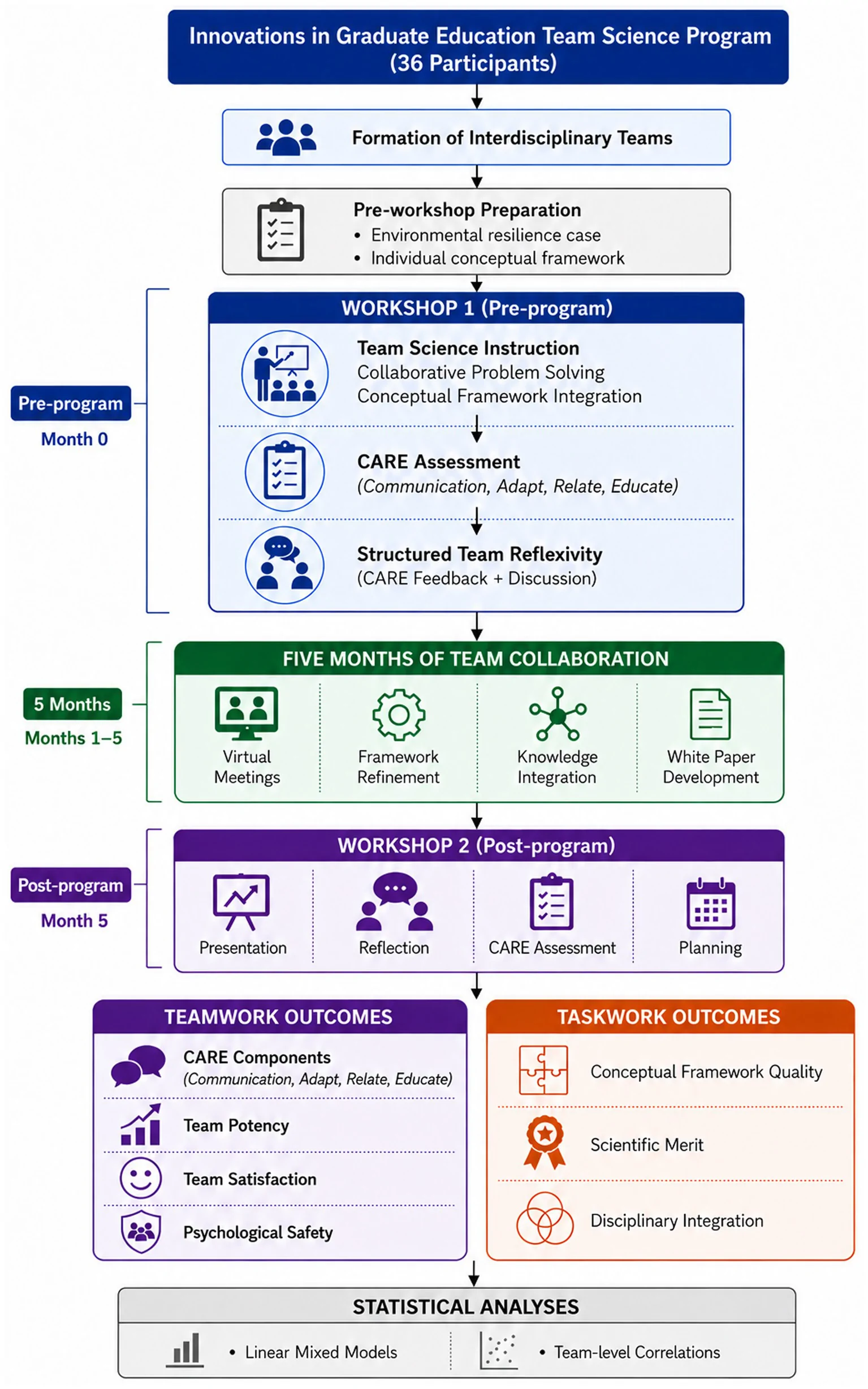
Trandisciplinary team science training.

#### 3.2.2 Workshop activities

During the first workshop, participants were given lectures by subject matter experts on topics relevant to transdisciplinary team science. This included instruction on the Science of Team Science, teamwork and group dynamics, and coastal resilience problems in the Chesapeake region. Interspersed were collaborative working sessions where participants worked with their teams on their assigned case. These sessions centered on their understanding of resilience in the context of their case, how they would go about studying this, and discussion of their conceptual frameworks. For the latter, participants used their individually developed conceptual frameworks as seeds to the initial collaborative problem solving. Team members were instructed to share their individual frameworks with team members, describe them to each other, engage in discussion about them, and finally, begin the work of trying to integrate these into a framework representative of their varied perspectives.

In addition to providing a structured problem context, the workshop incorporated reflection on their collaborative processes (described in more detail below). Faculty affiliated with the Virginia Institute for Marine Sciences were recruited to help guide students through their collaboration while not directly influencing their scientific thinking. Specifically, they took on more of a supportive role and worked to foster reflective practice. For this, they encouraged participants to consider what actions or interactions were contributing, or had contributed to effective collaboration processes.

### 3.3 Variables

#### 3.3.1 Teamwork

Our measures of teamwork consisted of the subcomponents of the Team CARE model (O’Neill et al., 2017), an assessment method that helps teams evaluate collaboration. Building off earlier measures of team effectiveness, O’Neill and colleagues developed and tested the CARE model for team development and were able to show how these are associated with team performance outcomes. It considers how individuals are communicating, adapting, reflecting, and educating each other while interacting. For example, “Communicate” and “Relate” dimensions assess the degree to which teams are able to articulate ideas or establish interpersonal trust. “Adapt” captures team responsiveness to new information and shifting conditions, while “Educate” reflects the degree to which members actively contributed to one another’s learning and development. CARE assessment has been used to provide feedback and improve team learning (O’Neill et al., 2018). The CARE survey has shown highly reliable psychometric properties, with published reliability coefficients typically falling within the acceptable to excellent range (e.g., α or intra-class correlations above 0.75; see O’Neill et al., 2017; O'Neill et al., 2019; O’Neill et al., 2020). As such, we used the CARE survey to help focus team member reflection, as well as assess how team processes evolved over the course of the training intervention. We additionally included a subset of questions assessing team attitudes potentially relevant to learning and performance. This included team potency (e.g., Guzzo et al., 1993), satisfaction with their team, and psychological safety (Edmondson, 1999). Survey items measuring team potency included statements such as, “No task is too tough for our team,” and “Our team believes it can solve any problem it encounters.” These items were intended to capture collective confidence and a sense of shared efficacy. Meanwhile, satisfaction items assessed preferences for continued collaboration, enjoyment of working together, and comparisons with other teams participants had experienced. High scores on these suggest a motivationally rich environment, which is critical for sustained engagement in complex research.

#### 3.3.2 Taskwork

The primary taskwork outcome was the quality of the transdisciplinary white paper produced by each team. This outcome was operationalized through independent expert ratings of multiple dimensions of white-paper quality. Each white paper served as a brief research proposal documenting what the team had learned about its case, the research needed to better understand the problem, and potential methods or interventions for addressing it. Teams were expected to integrate the disciplines represented among their members and incorporate a final conceptual framework illustrating the relationships among their key concepts.

We selected white-paper quality as the taskwork outcome because the product documents the team’s transdisciplinary scientific problem solving, requires an integrative conceptual framework, and represents an ecologically valid product of scientific collaboration. The dependent variables used in the analyses were expert ratings of the white papers. Specifically, to ensure ecological fidelity in evaluation, team white papers were separately reviewed by three subject matter experts (SMEs) from the University of Virginia, William and Mary, and the Virginia Institute of Marine Science. Collectively, these SME assessments provided performance measures that helped assess the integrative nature of the taskwork. To mirror the transdisciplinary scope of the student projects, the experts were selected from a diverse range of fields to ensure that the evaluation captured the interplay between ecological, technical, and socio-political dimensions of resilience. Collectively, these SME assessments provided performance measures that helped assess the integrative nature of the taskwork.

Scientific-merit ratings assessed novelty, advancement of knowledge, integration of ideas, and societal benefit. Because conceptual-framework development was a central component of the taskwork training, experts also rated the creativity, feasibility, and transformative potential of each framework (Table 1). Each of the questions shown were evaluated using Likert scales from 1 (strongly disagree) to 7 (strongly agree). The exception was the question: “Please rate the intellectual merit of the proposal, i.e., the potential to advance knowledge?” Because this did not readily lend itself to a Likert values, SMEs were asked to evaluate intellectual merit as poor, fair, good, very good, or excellent. Finally, experts provided an overall rating of disciplinary integration by responding to the following item on a 1–10 scale: “To what degree did the research proposal achieve integration across the disciplines represented on the team?” (Table 2).

**Table 1:** White Paper evaluation items for conceptual framework and sceintific merit.

Conceptual Framework: This project is funded by an NSF Innovations in Graduate Education grant that is testing, among other things, the use of visualization tactics to facilitate integration of knowledge from different disciplines.	
Creative	Does the conceptual framework explore creative concepts or relationships?
Feasible	Is the conceptual framework specific enough to develop feasible research questions?
Transformative	Does the conceptual framework present potentially transformative concepts?

Scientific Merit: From the perspective of a technical reviewer for a funder, please consider the scientific merit of the proposed research.	
Intellectual Merit	Please rate the intellectual merit of the proposal; that is, the potential to advance knowledge.
Novelty	Is this an important, novel research question?
Integration	Do the preliminary research methods show novel approaches, informed by an integrated perspective?
Societal Benefit	Will the proposed activity benefit society, have broad societal impact?

**Table 2:** Evaluation of White Paper integration of scientific disciplines represented in their team.

Degree of integration									
Multidisciplinary		Interdisciplinary				Transdisciplinary			
Each discipline makes an independent, additive contribution.		Integration of methods and perspectives from two or more disciplines. Synergies beyond additive.				Integrates and transcends disciplinary boundaries. Fundamentally novel frameworks, theories, models, methods.			
1	2	3	4	5	6	7	8	9	10

### 3.4 Team learning intervention

Although not manipulated as an independent variable, our team science intervention was team reflexivity. As described earlier, reflexivity helps teams develop a general competence about their teamwork by learning how to reflect on components of collaboration. To be effective, team reflections need to be based on feedback using evaluation of their collaboration. Feedback is crucial because it provides a focus for team discussion. Teams were instructed to use their feedback from CARE to reflect on team processes and to then identify potential strategies for improvement.

Within our workshop structure, reflexivity was developed as a key mechanism for supporting learning in teams. Teams were coached to review feedback from their CARE assessment, reflect on this in the context of their scientific problem solving to interpret accomplishments and failures, and plan future action. We emphasized “thinking together” about both outcomes and processes, thereby enhancing the team’s ability to learn and adapt. Teams were prompted to engage in structured discussions around key questions based upon their responses to the sub-components of the CARE model (see Table 3 for definition of CARE subcomponents and associated reflection prompts). These discussions enabled teams to generate shared meaning while articulating methods for improvement. In this way, reflexivity is integrated with their team problem solving sessions to support their ability to self-correct and adjust to complexity and uncertainty in their teamwork and taskwork.

**Table 3:** Example of team sheets used to guide reflection on their CARE assessement feedback. Note the blank cells expanded to allow as much writing as teams desired.

*Instructions: As a team, please complete the following reflection activity. Use the “Team Health” report provided to understand what your team-level response is for each dimension. Be very specific with your action steps.*	
Communicate dimension	Action steps: List steps and actions that your team will take to improve each dimension
Cooperative conflict management: Approaching conflict and incompatibilities such that win-win solutions are sought. Conflict is viewed as a chance to learn and make quality improvements, and members work through different viewpoints with mutual respect.	
Role clarity: The existence of well-defined and understood roles within the team, demonstrated through the establishment and maintenance of clarity regarding responsibilities, goals, expectations, and relative authority of each role within the team.	
Strategy formulation and planning: Developing overall strategies that guide team efforts, including the sequencing of planned work elements, evaluation of processes, and formulation of contingency plans.	

*Instructions: As a team, please complete the following reflection activity. Use the “Team Health” report provided to understand what your team-level response is for each dimension. Be very specific with your action steps.*	
Adapt dimension	Action steps: List steps and actions that your team will take to improve each dimension
Team monitoring and backup: A shared awareness of the relative strengths and weaknesses of each team member, supplemented by an effort of all members to monitor work standards and offer help to each other when needed.	
Goal progression: The regular evaluation by team members of the progress and accomplishment of team goals. Team members use clearly defined metrics to assess progress, pacing, and accomplishment of goals.	
Coordination: Smooth workflow between team members, integration and coordination of each team member’s work efforts, and efficient re-establishment of coordination in response to error.	

*Instructions: As a team, please complete the following reflection activity. Use the “Team Health” report provided to understand what your team-level response is for each dimension. Be very specific with your action steps.*	
Relate dimension	Action steps: List steps and actions that your team will take to improve each dimension
Contribution equity: Agreement in the adequacy of each member’s contribution to the team effort and objectives.	
Healthy, fact-driven conflict: The team freely and openly debates the merits of different perspectives, views, and opinions on an intellectual level.	
Lack of personal conflict: The team atmosphere is free of interpersonal tensions, friction, animosity, and personality clashes.	
Trust: Extent to which team members perceive and have confidence in the integrity, reliability, and overall trustworthiness of members within their workgroup.	

*Instructions: As a team, please complete the following reflection activity. Use the “Team Health” report provided to understand what your team-level response is for each dimension. Be very specific with your action steps.*	
Educate dimension	Action steps: List steps and actions that your team will take to improve each dimension
Constructive controversy: The development and expression of all members’ ideas and an effort to understand and question them; the integration of unique components of ideas; an assurance of unanimous agreement prior to the implementation of a decision.	
Exploitative learning: Efficiency-enhancing behavior that involves refining, capitalizing on, and leveraging existing team member knowledge to complete tasks and move forward on deliverables.	
Exploratory learning: Searching for new information beyond the team’s current knowledge base with a focus on flexibility, discovery, and open-mindedness toward new concepts and possibilities.	

Data were collected using the CARE instrument across a two-phase workshop series spanning 5 months. The procedure followed a longitudinal pre-post design tailored to two specific collaborative events: the initial workshop held on the Eastern Shore of Virginia with a follow-up session in Virginia Beach approximately 5 months later. Participants completed the CARE assessment and reflexivity exercise at four distinct time points to capture the evolution of team dynamics relative to key project milestones: Time 1 and Time 2: Data were collected during the initial workshop. The first was following the team’s initial collaborative problem solving session (Time 1) at the end of the first day, and immediately upon the conclusion of the Eastern Shore workshop (Time 2). Between these two intervals, teams engaged in intensive remote interaction, dedicating hours to individual scientific problem-solving and also working as virtual teams to collectively synthesize their initial shared conceptual models. Time 3 (Time 3): Administered at the beginning of the second workshop approximately 5 months after the first, capturing team dynamics following the interim work period between November and March, and just prior to the teams’ formal presentation of their conceptual models. Time 4 (Time 4): Collected at the conclusion of the in-person Virginia Beach session. This final assessment allowed teams to reflect on their workshop performance and presentation, serving as a planning step for the final performance period, which extended beyond the end of the workshop. For the purposes of this paper, we only analyze the initial and ending data collection (Time 1 and Time 4) to understand the overall impact of the workshop. We will refer to these as Pre-program and Post-program (pre and pro) in the remainder of the paper.

### 3.5 Analysis plan

We analyzed survey data collected at two training time points. First, to establish baseline perceptions of working with newly formed teams, and, second, following the final workshop, to assess perceptions after the overall training experience. Separate linear mixed-effects models were estimated for each CARE component so that each retained its own fixed-effect estimates and random-effect variance. Prior to computing the CARE composite scores, two participants had isolated missing responses on a small number of survey items. To avoid excluding these participants when constructing the composite variables, the missing item responses were replaced using the corresponding team mean. This preprocessing decision affected only a small number of item-level responses and was completed before fitting the mixed-effects models, which were subsequently estimated using maximum likelihood.

## 4 Results

Because we have a sample of only six teams, for our primary analyses we ran two-level models with team as a fixed effect. Specifically, in all cases, Level 1 was time, Pre-program versus Post-program, and Level 2 was participants, with team set as a between fixed effect, and participants set as random intercepts. In this way we can assess changes at the individual level while controlling for team-level differences but still determine differences for teams.

### 4.1 Team process improvement

We first analyzed the Communication elements of the CARE model. We averaged across the sub-category items of the survey completed during the first workshop and the final workshop. A linear mixed-effects model was used to examine changes in team communication from the start of training to the end of training. For ease of explanation, we will call this pre-program (pre) and post-program (post), with Time as a within-person fixed effect, Team as a between-team fixed effect, and a random intercept for participants. We find a significant main effect of Time, *F*(1, 35) = 153.17, *p* < 0.001, indicating that Communication scores increased substantially from pre to post. We also find a significant main effect of Team, *F*(5, 30) = 2.56, *p* < 0.05, indicating that the six teams differed in their overall self-reported levels of Communication. Next, a linear mixed-effects model was used to examine whether Adapt changed from pre to post, accounting for repeated measures nested within individuals. Results revealed a statistically significant and large effect of Time, *F*(1, 35) = 143.04, *p* < 0.001, showing that Adapt scores increased substantially from pre to post. The effect of Team was not statistically significant, *F*(5, 30) = 1.85, *p* = 0.13.

A linear mixed-effects regression also examined changes in the Relate items over time, with random intercepts for individuals and fixed effects of Time and Team. There was a significant main effect of Time, *F*(1, 35) = 29.13, *p* < 0.001, indicating that Relate scores increased substantially from pre to post with the effect of Team also significant, *F*(5, 30) = 3.12, *p* = 0.022, indicating between-team variation. A linear mixed-effects model examined whether the Educate behaviors changed from pre-test to post-test while accounting for repeated measures nested within participants. There was a significant effect of Time, *F*(1, 35) = 169.47, *p* < 0.001, suggesting that Educate scores increased substantially from pre to post, but the effect of Team was not statistically significant, *F*(5, 30) = 1.03, *p* = 0.42.

Overall, as shown in Figure 2, these results demonstrate a large and robust improvement in components of CARE over the course of the teams professional development. The significant main effects of Time indicate that, on average, participants reported improvements across the training period.

**Figure 2:**
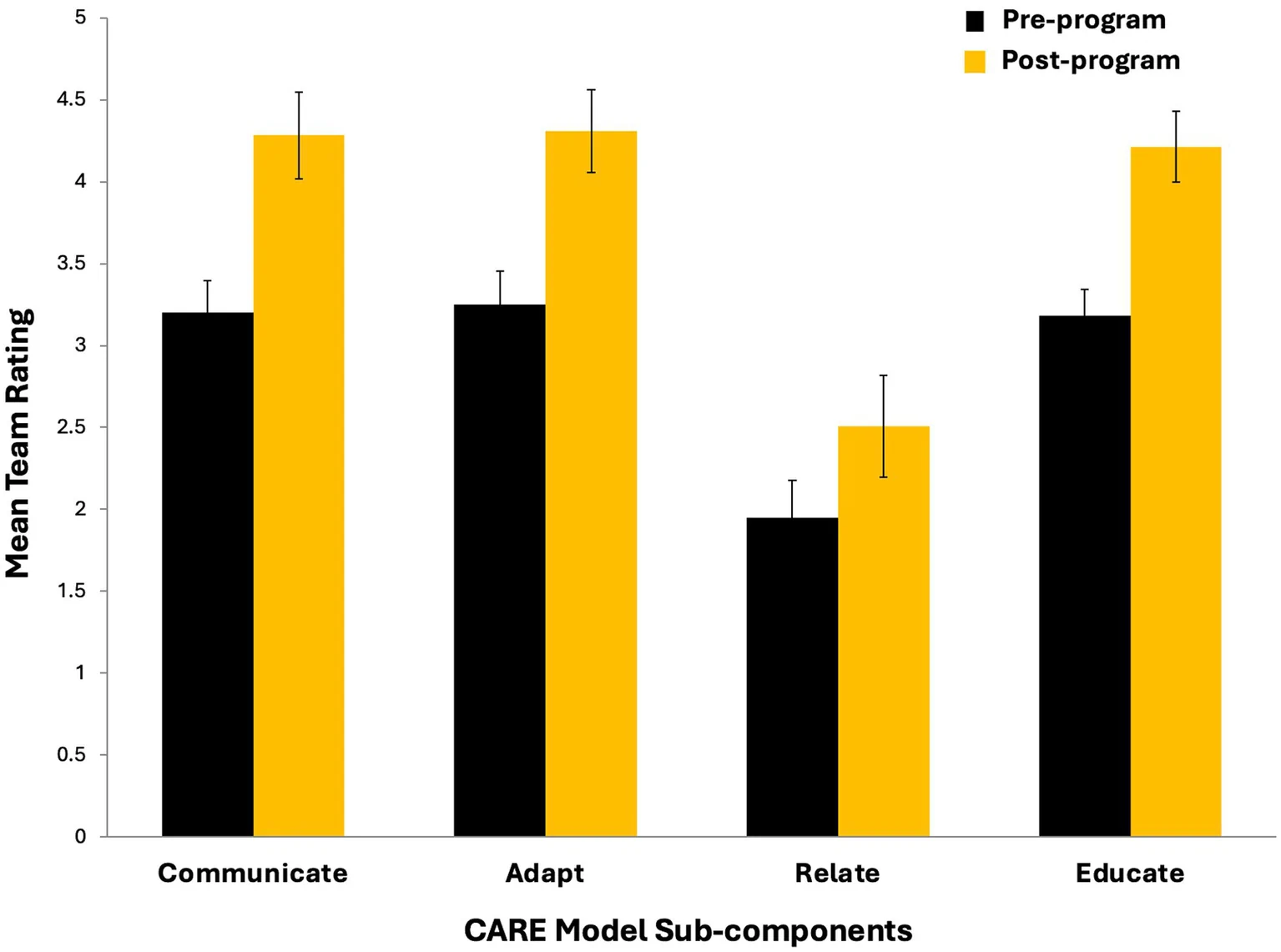
CARE model changes from start to ending of workshop experience.

### 4.2 Assessing team attitudes

As described, subjective indicators of concepts related to team attitudes were also examined to assess how participants experienced their collaborative work. This included Team Potency, Team Satisfaction, and Psychological Safety.

A linear mixed-effects model examined whether team potency changed from pre-test to post-test. There was a significant effect of Time, *F*(1, 35) = 43.41, *p* < 0.001, with potency increasing from pre to post. The Team effect was not significant, *F*(5, 30) = 1.29, *p* = 0.293.

A linear mixed-effects model also evaluated changes in team satisfaction across time with random intercepts for participants. There was a significant effect of Time, *F*(1, 35) = 139.65, *p* < 0.001, indicating that team satisfaction increased substantially from pre to post. Team satisfaction also differed significantly among teams, *F*(5, 30) = 2.57, *p* = 0.048.

Last, a linear mixed-effects model evaluated changes in psychological safety across time. There was no significant effect of Time, *F*(1, 65) = 0.71, *p* = 0.404, with psychological safety remaining high and stable from pre to post. The Team effect was also nonsignificant, *F*(5, 65) = 1.77, *p* = 0.131. Thus, psychological safety did not appear to change because of the training and showed minimal between-team variability.

### 4.3 Summary

Across the four components of the CARE model, and two of the three team attitudes measures (team potency and team satisfaction), there were consistent and substantial improvements from pre-test to post-test, demonstrating increases in perceptions of their team functioning. In contrast, psychological safety was already high at baseline and showed little room for change. Overall, the multilevel results indicate improvement in core teamwork processes and attitudes, with small but occasionally meaningful differences between teams.

## 5 Evaluating collaborative research outputs

Team white papers were developed to codify research ideas around the problem they were given. This was developed as a capstone to the experience where they articulated their ideas behind their conceptual framework for resilience, while also outlining a proposed research agenda. As described, three subject matter experts (SMEs) evaluated the white papers to help assess the integrative nature of the taskwork. Inter-rater reliability across the three SMEs was assessed using the Intraclass Correlation Coefficient (ICC) based on a two-way random-effects model with absolute agreement. The analysis indicated moderate reliability for the average of the three evaluators, ICC(2,3) = 0.54.

### 5.1 Process improvement and scientific team output

As described, white papers were evaluated on dimensions of scientific merit mirroring traditional grant proposals at funding institutions as well as with evaluation of their conceptual frameworks along dimensions of creativity, feasibility, and transformative ideas. Last, there was an overall assessment of disciplinary integration.

We next show the associations between team process variables and expert evaluations of the scientific white papers. Because analyses were based on only six teams, we examined both Pearson and Spearman correlations. The pattern of associations was similar across methods; therefore, we report Spearman correlations because they are more robust to small sample sizes and departures from normality. Given the small sample of six teams, these analyses are intended to identify patterns of association rather than provide definitive statistical inference.

We correlated our team process predictors, including the change scores, with our science team output variables. That is, the assessments from the CARE model, along with subjective team assessments were correlated with expert evaluations of the white papers. This analysis explored the relationship between science team processes over time and the visual and semantic conceptual quality of their scientific output. Table 4 shows the descriptives for the SME ratings and Table 5 provides Sperman correlations for the six teams on the SME ratings, with color shading indicating strength of the association.

**Table 4:** Means and standard deviation of white paper evaluation items.

	Disciplinary integration	CF creative	CF feasible	CF transformative	SM advance knowledge	SM novelty	SM integrated	SM societal benefit
Mean (SD)	5.67 (1.34)	5.33 (0.65)	5.11 (0.67)	4.67 (0.80)	3.50 (0.64)	5.33 (0.80)	4.94 (0.60)	5.11 (0.61)

**Table 5:** Correlations between team process variables difference scores and subject matter expert ratings of white paper quality.

Team disciplinary integration	Team disciplinary integration	CF creative	CF feasible research	CF transformative	CF average	SM advance knowledge	SM novel	SM integrated	SM societal benefit	Scientific merit average
Starting time (Pre)										
Pre communicate	0.09	**−0.91**	**−0.75**	**−0.85**	**−0.93**	**−0.76**	**−0.72**	**−0.76**	**−0.66**	**−0.75**
Pre adapt	0.12	**−0.93**	**−0.71**	**−0.79**	**−0.89**	**−0.75**	**−0.62**	**−0.75**	**−0.65**	**−0.71**
Pre relate	**0.75**	**−0.41**	−0.09	**−0.53**	**−0.49**	−0.06	−0.26	−0.06	0.00	−0.09
Pre educate	−0.06	**−0.99**	**−0.77**	**−0.88**	**−0.94**	**−0.81**	**−0.71**	**−0.81**	**−0.74**	**−0.77**
Pre team potency	**0.53**	**−0.74**	**−0.41**	**−0.58**	**−0.67**	**−0.46**	−0.31	**−0.46**	−0.34	**−0.41**
Pre team satisfaction	0.23	**−0.58**	−0.09	−0.18	−0.26	−0.23	0.18	−0.23	−0.15	−0.09
Pre psychological safety	−0.31	**−0.79**	**−0.78**	**−0.68**	**−0.78**	**−0.79**	**−0.68**	**−0.79**	**−0.68**	**−0.78**
Ending time (Post)										
Post communicate	−0.09	0.20	0.03	0.09	0.09	0.12	−0.09	0.12	0.21	0.03
Post adapt	**0.55**	0.09	−0.09	0.00	−0.09	−0.03	−0.09	−0.03	0.03	−0.09
Post relate	**0.75**	**0.52**	**0.60**	0.35	**0.43**	**0.64**	**0.44**	**0.64**	**0.59**	**0.60**
Post educate	0.06	0.12	−0.09	−0.09	−0.09	0.03	−0.26	0.03	0.12	−0.09
Post team potency	0.32	**0.55**	**0.49**	0.26	0.37	**0.61**	0.18	**0.61**	**0.65**	**0.49**
Post team satisfaction	0.12	0.35	0.26	**0.53**	**0.43**	0.23	**0.44**	0.23	0.29	0.26
Post psychological safety	0.03	−0.03	**−0.43**	−0.35	−0.37	−0.29	**−0.62**	−0.29	−0.26	**−0.43**
Difference scores (Δ = Post-program – Pre-program)										
Δ Communicate	−0.17	**0.41**	0.26	0.26	0.31	0.35	0.09	0.35	**0.41**	0.26
Δ Adapt	0.06	**0.41**	0.03	**0.44**	0.31	0.06	0.18	0.06	0.09	0.03
Δ Relate	0.35	**0.81**	**0.71**	**0.53**	**0.66**	**0.81**	**0.44**	**0.81**	**0.77**	**0.71**
Δ Educate	0.20	0.32	0.26	0.26	0.26	0.32	0.18	0.32	**0.41**	0.26
Δ Team Potency	−0.26	**0.72**	**0.54**	**0.44**	**0.60**	**0.64**	0.26	**0.64**	**0.56**	**0.54**
Δ Team Satisfaction	−0.12	**0.81**	0.31	**0.79**	**0.71**	0.35	**0.44**	0.35	0.29	0.31
Δ Psychological Safety	0.12	0.23	−0.14	−0.09	−0.09	0.00	−0.35	0.00	0.03	−0.14

Spearman’s rank correlations (ρ) are reported for six teams. Emphasis is placed on effect sizes rather than statistical significance because of the small sample size. Given the small sample, these coefficients should be interpreted as exploratory rather than confirmatory statistical tests. Bold coefficients indicate |ρ| ≥ 0.40. Cell shading indicates effect size magnitude: dark green = |ρ| ≥ 0.85; medium green = 0.70–0.84; light green = 0.40–0.69. CF=Conceptual Framework; SM = Scientific Merit.

The most consistent correlation with proposal quality was change in relational processes (i.e., difference scores for the average across the Relate items of the CARE model). Improvements in Relate were positively associated with proposal creativity, feasibility, scientific integration, societal benefit, and overall scientific merit, with most correlations ranging from *ρ* ≈ 0.71 to 0.81. These findings suggest that teams demonstrating the greatest growth in processes like healthy, fact-driven conflict and contribution equity also produced proposals judged to be of higher overall quality. Although changes in Adapt were associated with proposal quality in the Pearson analyses (i.e., ratings of transformative impact and novelty associated with Adapt), these relationships were weaker using Spearman’s rank correlations. This indicates that they were less robust to the small sample and should therefore be interpreted cautiously. Similarly, positive associations with Team Satisfaction and proposal quality found in correlations with white paper transformativeness and novelty differed when using the Spearman analyses. This suggests these variables may also have only modest relationships with proposal evaluations.

In contrast, several baseline (pre) process variables demonstrated consistent negative associations with proposal quality across both Pearson and Spearman analyses. Baseline Communication, Adapt, and Educate scores were negatively associated with proposal creativity, transformative potential, conceptual-framework quality, and scientific merit, with correlations typically ranging from *ρ* ≈ −0.70 to −0.95. These findings suggest that teams entering the program with higher initial ratings on these process dimensions did not necessarily produce the strongest scientific proposals. Rather, the strongest proposals were associated with teams that demonstrated greater development in Relate related functioning over the course of the program.

The teams that ultimately produced the best proposals were not necessarily the teams that looked strongest at the outset. Instead, they were the teams that changed the most in their relational processes. This fits a developmental view of team science, where improving in their relational capacity during collaboration is more consequential than initial perceptions of teamwork. Together, these findings suggest that how teams developed during the collaboration may have been more important than where they began.

Considering these findings alongside the teams’ conceptual frameworks provides additional insight into the mechanisms underlying successful collaboration. The highest-performing teams produced conceptual frameworks that demonstrated greater coherence, disciplinary integration, and conceptual organization, suggesting that these visual cognitive artifacts supported the development of shared understanding while simultaneously improving collaborative processes. These attributes were likely supporting not only the exchange of disciplinary knowledge but a restructuring of their own mental models to foster more integrative problem solving (e.g., Clark and Chalmers, 1998; Fiore et al., 2010; Fiore and Schooler, 2004; Fiore and Wiltshire, 2016). In contrast, lower-performing teams tended to produce fragmented conceptual frameworks that lacked an overarching structure for integrating disciplinary perspectives, limiting opportunities for effective knowledge coordination and synthesis. Specifically, they suggest that improvements in collaborative processes like relational functioning can be linked to the development of higher-quality conceptual frameworks as well as more effective scientific products. Overall, these comparisons reinforce the importance of studying teamwork and taskwork (conceptual frameworks) and how these relate to team science effectiveness.

## 6 Discussions

This study described a workshop-based program of professional development in transdisciplinary teamwork. Participants collaborated on teams composed of different disciplines but united in the study of coastal resilience. Our findings provide several important contributions to the Science of Team Science literature. First, we provide one of the first team level assessments of scientific team learning. Specifically, we assessed team process and outcomes aggregated to the level of the team. The vast majority of prior studies of team science correlates individual level responses without aggregation to the team level. Second, this was one of the first science team training studies to directly link team level processes to objective measures of scientific outcomes. Third, related to these, an important unique contribution was our complementary use of teamwork and taskwork concepts. Fourth, this allowed for assessment of teamwork and taskwork in the context of the teams’ transdisciplinary research product. Finally, overall, this study is one of the first research programs in the Science of Team Science to systematically introduce instruction, practice, and feedback focusing on teamwork. As noted at the beginning, because scientific training rarely includes explicit instructions on how to work on a team and/or provide feedback on teamwork, it is crucial for this to be included in educational practice (Graesser et al., 2018). We provide a model for building these kinds of best practices for improving science overall.

We developed this approach based upon distinctions coming out of the organizational sciences on team training (Cannon-Bowers et al., 1995; Salas et al., 2000). Specifically, effective teams, including teams of scientists, must engage in both taskwork and teamwork. This distinction is crucial to understand team effectiveness because team members could each be highly competent and effective at their scientific tasks, but, without effective teamwork, the team, as a whole, could fail in their collaborative scientific problem solving.

The integration of these two overarching concepts was foundational to our workshop and assessment. We assessed how the effective integration of these within a team drives research outcomes. We measured the development of team competencies as well as scientific taskwork over the course of their training. For teamwork, we used a robust method of collaboration that considered a core set of team processes (i.e., CARE model assessment and reflection). For taskwork, we used subject matter experts to evaluate the research product produced by the teams. This was essentially an external peer review on the transdisciplinary research proposals that were developed by the teams. In addition to more traditional research proposal criteria, such as the scientific and technical merit, we also included perceptions on knowledge integration via their conceptual framework. The aforementioned evaluations assess how well teams cover the problem space associated with their resilience challenge problems (cases) and engage in knowledge integration while doing so. From this we were able to assess the degree to which concepts are connected in their research plans. Again, this was based on evaluation of the conceptual frameworks students produced. As described, these are cognitive artifacts co-created as graphical visualizations developed over the course of the training (e.g., Fiore and Wiltshire, 2016; Hollan et al., 2000).

In combination, our measurement of teamwork processes now adds scientific collaboration to the general team literature using self- and peer evaluation of collaboration (cf. Loughry et al., 2007; O’Neill et al., 2017, 2020; Schippers et al., 2003). We go further with inclusion of taskwork using traditional measures of research quality (proposal evaluation) and problem space coverage and integration, providing a useful guide for understanding how workshops can affect transdisciplinary learning and research outcomes.

### 6.1 Triangulating team performance: CARE, SME ratings, and subjective experience

The integration of self-reported team process measures (via the CARE model), expert evaluations of research outputs, and subjective assessments of team attitudes provided a comprehensive evaluation of the workshop intervention and a multidimensional view of science team performance. Together, these measures demonstrated how internal team processes relate to external indicators of scientific outcomes.

Although caution is warranted given the small sample, our analyses showed how different scientific outcomes were associated with different team processes. Growth in the ability to relate as a team was associated with scientific quality, suggesting the importance of interpersonal understanding for science teams. Conceptual framework quality was associated with attitudes like team satisfaction and potency. This suggests that the production of quality scientific products improves team member self-perceptions of the team and confidence in performance. In the context of transdisciplinary teamwork, this suggests satisfaction with their team’s ability to integrate diverse research concepts (e.g., how spawning frequency is related to economic outcomes in local fishing towns, and how interventions to limit fishing season length may differentially affect population growth dependent upon species).

The CARE framework also proved valuable for assisting with formative assessment. By using this to provide team feedback, it helped focus reflective processes and identify ways to improve their teamwork. Further, by tracking these longitudinally, it enabled comparisons across teams and demonstrated that improvements in communication, responsiveness to feedback, interpersonal relationships, and mutual learning accompanied stronger conceptual integration and higher-quality scientific outputs. These findings provide a foundation for further research examining how collaborative structured reflection can improve scientific team development (Schaffner et al., 2016).

More broadly, this study contributes to the Science of Team Science (SciTS) by demonstrating that concepts and measures from the general team literature can be successfully applied to scientific collaborations. CARE captures well-established antecedents of team effectiveness, integrating interpersonal processes with learning and adaptation (O’Neill et al., 2018). Our findings support socio-cognitive perspectives on teamwork, showing that successful collaboration emerges through the interaction of cognitive processes, social relationships, and externalized knowledge representations rather than from the aggregation of individual expertise alone (e.g., Pennington et al., 2016; Salazar et al., 2012). These findings provide useful insights for team science research and training. Interventions that strengthen relational awareness, reflection, and knowledge integration may improve collaborative processes and scientific quality. More broadly, these collaborative competencies (i.e., teamwork) need to be cultivated alongside disciplinary expertise (i.e., taskwork). Future longitudinal research should examine how these competencies develop over time and how conceptual modeling, and structured reflection can further enhance science team effectiveness.

### 6.2 Limitations

Despite contributing to the empirical base for research on scientific teams, a number of limitations should be considered when interpreting these findings. Most notably, the study involved a small number of teams (*N* = 6). Additionally, a small amount of item-level missing data was handled through mean substitution prior to scale construction. This approach retained participants in an already small sample, but alternative approaches such as multiple imputation or person-mean substitution may be preferable in future studies. Although the intensive, field-based design provided rich ecological validity, the limited number of teams constrained statistical power, particularly for team-level correlational analyses linking process change to expert-rated scientific outputs. As such, inferential statistics for these analyses should be interpreted as suggestive rather than definitive. In addition, participants were self-selected graduate students, which may limit generalizability to other scientific teams, such as faculty-led, industry-based, or high-stakes research collaborations operating under different incentive structures. Further, there was no control or comparison group, with all participating teams receiving the same teamwork and taskwork training. As such, this study relied only on change scores over the course of the professional development.

The study also relied primarily on self-reported measures raising concerns about common-method variance and social desirability effects. Although repeated measurement and multilevel modeling help address these issues, observed improvements may partially reflect increased awareness of teamwork concepts rather than sustained behavioral change. Additionally, the absence of direct interaction-based measures limits insight into the specific mechanisms linking reflexivity to particular processes (e.g., conceptual framework development) and improved collaboration.

Future research would benefit from adaptations to larger, multi-site samples, or inclusion of experimental or quasi-experimental designs that disentangle intervention elements. Additional work could include longitudinal follow-up assessing whether observed gains in teamwork translate into longer-term scientific productivity. Despite these constraints, the study makes a useful contribution by demonstrating, in an ecologically valid field setting, that theoretically grounded taskwork and teamwork processes are systematically related to the quality of transdisciplinary scientific outputs at the team level.

## 7 Conclusion

### 7.1 Linking process, product, and impact in team science

This field study demonstrates that integrating behavioral, cognitive, and outcome measures provides a more comprehensive understanding of science team effectiveness than relying on traditional research outputs alone. Teams producing the strongest conceptual frameworks and highest-quality white papers also demonstrated stronger relational processes, reinforcing the close association between how teams work and what they produce. These findings highlight the value of combining expert evaluations with process measures and structured reflection to assess both team development and scientific performance.

More broadly, the study contributes to the growing body of empirical work by providing a robust method for introducing established concepts from the general team literature within the Science of Team Science (SciTS). By linking team processes to measurable scientific outcomes, the CARE framework offers both a practical assessment approach and a mechanism for focusing reflective processes.

### 7.2 Implications for broader scientific communities and institutions

The findings suggest that collaborative competencies should be developed alongside disciplinary expertise in graduate education, postdoctoral training, and professional development. Institutions and funding agencies should likewise recognize that effective scientific teamwork is not simply a byproduct of assembling talented individuals but based on collaboration competencies that can be intentionally developed and evaluated.

Future research should pursue longitudinal and mixed-methods studies to determine how collaborative processes influence sustained scientific productivity, innovation, and societal impact over time and contexts.

Ultimately, this research reinforces one of the central premises of the Science of Team Science, that is, that scientific breakthroughs depend on both taskwork and teamwork (Fiore, 2008). Specifically, science team effectiveness draws from both the expertise individuals bring to a team as well as the collaborative competencies enabling expertise to be integrated into new knowledge. Understanding, measuring, and strengthening those processes will be essential to advancing transdisciplinary science and addressing increasingly complex societal challenges.

## Data Availability

The datasets presented in this article are not readily available because the data is still being analyzed by the research team for further manuscripts and, as such, is not yet sharable. Requests to access the datasets should be directed to Stephen Fiore, sfiore@ucf.edu.
